# Effects of an 8-week pre-season targeted training on sprinting performance, agility and lower limb muscular asymmetries in elite soccer players

**DOI:** 10.5114/biolsport.2024.134754

**Published:** 2024-03-06

**Authors:** Artur Gołaś, Przemysław Pietraszewski, Robert Roczniok, Artur Terbalyan, Adam Maszczyk, Rafał Opaliński, Adam Zając

**Affiliations:** 1 Institute of Sport Sciences, The Jerzy Kukuczka Academy of Physical Education, Katowice, Poland; 2 InnoGIO, Warszawa, Poland

**Keywords:** Soccer, EMG, Running speed, Muscle patterns, Strength

## Abstract

The purpose of this study was to determine the effects of an 8 week targeted training program on speed, agility, and muscle asymmetries in soccer players. 32 elite soccer players were recruited for the study. Their age, body mass, and body height were 25.8 ± 7.3 years, 77.4 ± 11.1 kg, and 177.5 ± 9.8 cm, respectively. After the warm-up, participants performed two maximum 30 m sprints with a 5-minute rest interval between trials. After the linear sprint test, performed two repetitions of the COD randomized ZigZag test. The tests were performed at the beginning and at the end of the pre-season, which lasted for 8 weeks. EMG was measured bilaterally from the quadriceps, hamstrings, and gluteal muscles with shorts made of knitted fabric similar to elastic clothes. Athletes in the experimental group performed sport-specific targeted strength training based on movement patterns 4 times per week (Monday, Tuesday, Thursday, and Friday). The training included 6 bodyweight exercises (Bird Dog, Single-Leg Glute Bridge, Dead Bug, Side Plank, Reverse Lunge, and Clamshell), performed unilaterally in 5 sets of 10 repetitions of each exercise. The load progression included an additional set in each successive week of the experiment. The data analysis showed significant improvement in 5 m and 30 m sprints after applying the 8-week targeted training protocol. A statistically significant improvement in performance was also observed after the 8-week training period in case of COD, while the speed deficit also decreased significantly. The greatest improvements were observed during the COD test. As a result of repeated COD training over a period of 8 weeks, its technique was improved. Implementation of training methods, which target specific aspects of soccer in the pre-season training routines can improve key motor abilities for that sports discipline. A comprehensive training approach including speed, agility, and resistance training based on specific movement patterns should be applied by strength and conditioning practitioners in soccer teams to achieve peak physical performance and reduce injury risk due to the partial elimination of muscular asymmetries.

## INTRODUCTION

The game of soccer is constantly evolving. In terms of physical requirements, the game has become significantly faster. For example, statistics from the English Premier League indicate that players at all positions are covering greater distances, at higher speeds, than ever before. Often, a player performs 50 or more sprints in a match, and these actions can play a vital role in determining the outcome of the game [1, 2]. Players are also required to change direction more than a thousand times per game, or approximately every six or seven seconds. For modern players, speed and agility are two key components that contribute to the ultimate quality of performance [3, 4, 5]. Typical high-speed movements are performed in soccer within seconds and usually over a distance of 5 to 30 m. Considering the relatively short distance of soccer sprints it seems logical that coaches concentrate on developing acceleration rather than maximum sprinting speed. The ability to change direction (COD) while running at high speed is of great significance in team sports, such as soccer, because it better reflects match conditions [5, 6, 7, 8]. In football, the significance of preseason preparations cannot be overstated. Teams engage in rigorous training sessions, focusing on enhancing players’ fitness, tactical understanding, and teamwork. These preparations lay the foundation for a successful season, helping players build chemistry, adapt to new strategies, and minimize the risk of injuries. Coaches meticulously plan drills, analyze opponents, and fine-tune strategies to ensure their squad is well-prepared for the challenges ahead. The preseason serves as a crucial time for players to sharpen their skills, cultivate cohesion, and foster a winning mentality that can propel them through the upcoming season.

The general adaptation syndrome (GAS) is one of the most significant principles applied in sports training. GAS describes the process the body of an athlete goes through when it is exposed to any kind of stress, positive or negative [9]. In a different sense, adaptation means the adjustment of the athlete’s cells, tissues, organs and the whole body to the surrounding environment. If the environment changes, the organism also changes to survive and function under the new conditions. The training effects in the scientific literature are often presented in a two-dimensional manner, describing acute and delayed changes. Immediately after a training session, performance usually declines as a result of fatigue. An athlete should not expect to become stronger after 1 set of exercise or 1 training session. Exposing the athlete to systematic exposure to progressive training loads will allow for increased strength potential. From a practical point of view, there are four main variables that determine the course of the adaptation process: the size of stimuli, adjustment, specificity, and individualization.

In a nutshell, training loads can be dedicated to three types of stimuli. First of them – is overloading, in which the magnitude of the load is above the neutral level and positive adaptation can occur. The second one is sustaining, in which the load is in the neutral zone and the level of performance is maintained. The last type of stimulus is regenerative, in which the magnitude of the stimulus allows the implementation of repairing processes and provides opportunities for recovery [3].

Taking together, training adaptation can be classified by specific training effects into acute effects, chronic effects, cumulative effects, delayed effects, partial effects, and residual effects

Soccer matches require players to perform repetitive power-related activities such as sprinting, jumping, accelerating, decelerating, change of direction, and cutting interspersed with periods of low to medium-intensity activity (e.g., walking). Therefore, in soccer locomotion speed tests are most popular, e.g., covering a certain distance in a straight line (5-30 m) or with a change of direction [10]. The ability to COD while running at high speed is of great interest to soccer coaches and athletes as it reflects match conditions [7]. A measure that helps in assessing the isolated COD capacity is the COD deficit. The COD deficit corresponds to the difference in speed between the linear sprint and the task of an equal distance with a change of direction [11].

Analysis of movement patterns for optimizing athlete’s development seems vital. Surface electromyography (sEMG) is a diagnostic tool in the field of clinical neurophysiology used to analyse the function of motor entities of a selected muscle or muscle groups by means of surface electrodes during exercise or at rest [12]. The data collected from EMG analysis provides information on whether a muscle is active or not, and informs about the fatigue and the activation or deactivation of a given muscle [13]

The advantage of EMG is displaying the results with speed and load data, which offers the opportunities for identification of the most involved muscle groups during the exercise [12]. Development of textile electrodes embedded in clothing that does not require careful placement of sensors or wires that restrict mobility may represent a new solution to the limitations of conventional surface electrodes [14].

Once the imbalance is identified, an intervention opportunity appears in the form of redefining the movement pattern. Thus, the introduction of direct intervention (ACUTE EFFECTS) before training, in the form of activating the weaker side or muscle group can be possible. The second option can be planning and implementing targeted resistance training over a specified period of time (TARGETED TRAINING), allowing for reduced imbalances and thus for long term improvement of the movement pattern (DELAYED EFFECTS). Such an intervention should be used both as an “activation warm-up” before the main training units or as a separate training session.

Functional asymmetry of the lower limbs (side-to-side asymmetry) greater than 15% [15] may increase the risk of knee injuries [13]. The dominance of one side of the body over the other may result in differences in the strength of particular muscle groups. Studies of soccer players have shown differences in strength and flexibility between the dominant and non-dominant limbs [16], and biomechanical asymmetries between each limb [17]. It has been proven that muscle strength deficiencies and asymmetries of lower limb muscles increase the risk of thigh muscle injury [18].

It has been documented that the lack of functional asymmetry between the muscles of the right and left leg occurs for results low-er than 9% of their activity. When the difference in muscle activity between the right and left limbs or parts of the body is between 9% and 18%, a tendency for asymmetry can be observed. An asymmetry greater than 18% indicates a serious imbalance and its origin should be determined and corrected by appropriate measures to avoid [19].

Considering the above data, the purpose of this study was to determine the effects of an 8 week targeted training program on speed, agility, and muscle asymmetries in soccer players.

## MATERIALS AND METHODS

### Participants

32 elite soccer players were recruited for the study. Their age, body mass, and body height were 25.8 ± 7.3 years, 77.4 ± 11.1 kg, and 177.5 ± 9.8 cm, respectively. They were randomly divided into two 16-person control and experimental groups. The participants did not perform any strenuous exercises 48 hours prior to testing to avoid fatigue. The players were informed verbally and in writing about the procedures, possible risks, and benefits of the tests, and provided written consent before the start of the study. The study received the approval (nr.6/2021) of the Bioethics Committee at the Academy of Physical Education in Katowice, Poland.

### Testing Procedures

The study was carried out on a treadmill at a multifunctional hall in The Jerzy Kukuczka Academy of physical education in Katowice.

The experimental session was conducted between 9:00 and 11:00 a.m. The session was preceded by a warm-up protocol, which included 5 minutes of jogging, several upper and lower body exercises like push-ups, body weight squats and split squats, 5- and 20-m sprints, and COD sprints. The running times were recorded by two pairs of dual-beam Witty Gate photocells (Microgate, Bolzano, Italy) with a measuring precision of 0.01 s. After the warm-up, participants performed two maximum 30 m sprints with a 5-minute rest interval between trials. After the linear sprint test, the soccer players rested for an additional 5 minutes and then performed two repetitions of the COD randomized ZigZag test [20]. The tests were performed at the beginning and at the end of the pre-season, which lasted for 8 weeks.

### Training Procedures

The intervention consisted of an 8-week training program. Three types of training sessions were used: speed training, agility training, and targeted strength training. The speed session included 15 sprints over a distance of 30 m performed at submaximal or maximal intensity. The rest intervals between repetitions were 2 minutes. After 5 and 10 repetitions, the rest interval was extended to 5 minutes. The agility session included 15 30-m COD runs. The speed session was performed twice a week (Monday, and Thursday) while the agility session was performed 3 times a week (Tuesday, Wednesday, and Friday).

During this period athletes in the experimental group performed sport-specific targeted strength training based on movement patterns 4 times per week (Monday, Tuesday, Thursday, and Friday). The training included 6 bodyweight exercises (Bird Dog, Single-Leg Glute Bridge, Dead Bug, Side Plank, Reverse Lunge, and Clamshell), performed unilaterally in 5 sets of 10 repetitions of each exercise. The load progression included an additional set in each successive week of the experiment. Control group received regular circuit training with the same volume and intensity as the experimental group. In addition to speed, agility, and targeted strength training, the players carried out normal soccer training including technical and tactical drills. The training mentioned above was designed to reflect testing conditions as much as possible.

### Electromyography

EMG was measured bilaterally from the quadriceps, hamstrings, and gluteal muscles with shorts made of knitted fabric similar to elastic clothes used for sports activities or functional underwear, with the exception of the possibility to measure EMG from the skin surface (Myontec Ltd, Kuopio and Suunto Ltd, Vantaa, Finland; [21]). To measure the average rectified EMG signal, bipolar electrode pairs were located on the distal part of the quadriceps, hamstrings, and glutes. The reference electrodes were located longitudinally along the left and right lateral sides (over tractus iliotibialis) providing valid and repeatable data [22]. The electrodes located on the quadriceps muscles collected data from vastus lateralis, vastus medialis and rectus femoris muscles. The vastus intermedius muscle is located deeply under the other muscles and thus was not included. The electrodes located on the hamstring muscles gathered the signal from the biceps femoris, semimembranosus, and semitendinosus muscles. The electrodes located on the glute muscles recorded signals from the gluteus maximus and gluteus medius muscles. The EMG signals were measured in a raw form with a sampling frequency of 1000 Hz and a frequency band of 50 Hz - 200 Hz (-3 dB). The raw EMG signals were first rectified and then averaged over each 100 ms interval without overlapping. Therefore, 10 consecutive samples from the raw signal formed one rectified averaged data sample. This 10 Hz data was stored in ASCII format in the module memory, and uploaded to the PC using custom software. From the 10 Hz EMG data, ARV was calculated from a 1 s window during a stable torque signal. Six channels (three from each leg) were recorded. To ensure proper signal conduction, the electrodes were moisturized with tap water before putting on the shorts. When wetted, a membrane covering the electrodes prevents the electrode-skin interface from drying.

### Statistical analysis

Statistical analyses were performed using Statistica 13.1. The results are presented as means, standard deviations, and confidence intervals. Normality of the distribution was verified using the Shapiro-Wilk tests, while the Levene’s test was used to examine homogeneity of variance and the Mauchley’s test was used to verify sphericity assumption. The statistical differences were determined by conducting a two-factor analysis of variance. Effect sizes for main effects and interactions were determined using partial eta squared (η2) and Cohen’s d (d) for pairwise comparisons. Partial eta squared values were classified as small (0.01-0.059), moderate (0.06-0.137), or large (> 0.137). Cohen’s d was characterized as large (d > 0.8), moderate (0.5 ≤ d ≤ 0.8), small (0.2 ≤ d < 0.5), or trivial (d < 0.05). Additionally, percent changes with 95% confidence intervals (95%CI) were calculated. The p-value was set at < 0.05 for statistical significance.

## RESULTS

**FIG. 1:**
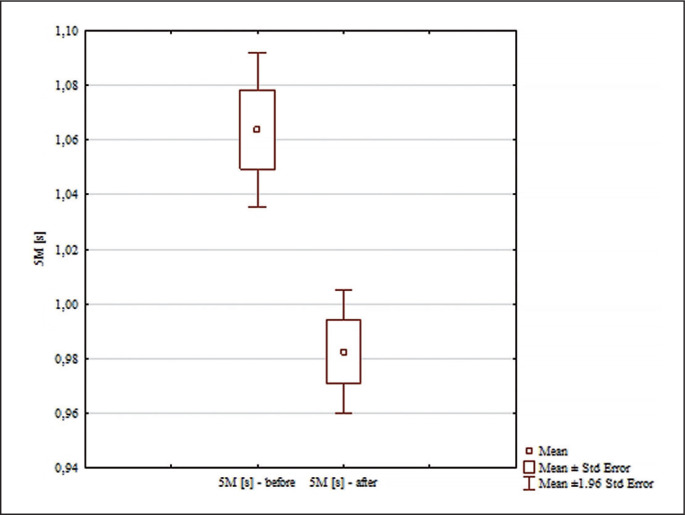
Comparisons between paired values for 5 m [s].

The analysis of the results in Table 1 and Table 2 allowed us to conclude that for all the variables there were no grounds to reject the null hypothesis of normality of the variables analyzed, so in subsequent analyses the statistics were based on parametric tools, namely analysis of variance with repeated measures and Tuckey’s post hoc multiple comparison tests. Analysis of the results for the 5 m [s] distance (Fig. 1) revealed significant differences for the main effects: differences between groups (F = 6.61; p = 0.015; ɳ^2^ = 0.18); differences for the main effects before and after (F = 93.89; p < 0.0001; ɳ^2^ = 0.75); and for the interaction group × measurement time (F = 15.85; p = 0.0004; ɳ^2^ = 0.35). To determine between which groups there were significant differences for the interactions, Tuckey’s post hoc multiple comparison tests were used. No significant differences were found between the results of the 5 m [s] test in the groups analyzed before the experiment after the experiment (p = 0.0023; d = 1.57). After the experiment, the time needed to run the distance was statistically significantly shorter in the experimental group than in the control (Fig. 1).

**TABLE 1:** Descriptive statistics for all measured variables, p-values for normality of the distribution

Variable	Experimental group		Control group	

	Pre	Post	Pre	Post

	M ± SD (-95%CI: 95%CI)	M ± SD (-95%CI: 95%CI)	M ± SD (-95%CI: 95%CI	M ± SD (-95%CI: 95%CI
5M [s]	1.064 ± 0.058 (1.03; 1.09)	0.98 ± 0.046 (0.96; 1.01)	1.078 ± 0.039 (1.058; 1.10)	1.044 ± 0.035 (1.025; 1.063)

30M [s]	4.24 ± 0.16 (4.15; 4.33)	4.08 ± 0.17 (3.98; 4.17)	4.24 ± 0.13 (4.16; 4.31)	4.19 ± 0.17 (4.10; 4.28)

Agility [s]	6.17 ± 0.29 (6.02; 6.33)	5.18 ± 0.29 (5.03; 5.34)	6.22 ± 0.19 (6.12; 6.32)	6.09 ± 0.24 (5.96; 6.22)

Deficit [s]	1.93 ± 0.24 (1.80; 2.06)	1.11 ± 0.21 (1.00; 1.22)	1.98 ± 0.13 (1.91; 2.05)	1.90 ± 0.15 (1.82; 1.98)

AQ	11.31 ± 1.89 (10.31; 12.32)	9.58 ± 1.36 (8.85; 10.30)	11.31 ± 1.85 (10.32; 12.29)	10.99 ± 1.84 (10.02; 11.97)

AH	18.04 ± 2.89 (16.50; 19.58)	13.66 ± 1.49 (12.87; 14.46)	17.90 ± 2.02 (16.82; 18.98)	17.25 ± 1.96 (16.20; 18.29)

AG	20.21 ± 4.11 (18.02; 22.40)	14.33 ± 3.26 (12.59; 16.06)	20.25 ± 3.61 (18.33; 22.17)	19.53 ± 3.40 (17.72; 21.34)

M – mean; Me ; SD – standard deviation, p S-W – p values for Shapiro-Wilk test, confidence intervals (95CI).

**TABLE 2:** Descriptive statistics for all measured variables, p-values for normality of the distribution

Variable	M	-95% CI	95% CI	SD	p S-W
5M [s] – before	1.064	1.03	1.09	0.058	0.42
5M [s] – after	0.98	0.96	1.01	0.046	0.44
30M [s] – before	4.24	4.15	4.33	0.16	0.50
30M [s] – after	4.08	3.98	4.17	0.17	0.30
Agility [s] – before	6.17	6.02	6.33	0.29	0.33
Agility [s] – after	5.18	5.03	5.34	0.29	0.21
Deficit [s] – before	1.93	1.80	2.06	0.24	0.51
Deficit [s] – after	1.11	1.00	1.22	0.21	0.62
AQ – before	11.31	10.31	12.32	1.89	0.15
AQ – after	9.58	8.85	10.30	1.36	0.29
AH – before	18.04	16.50	19.58	2.89	0.12
AH – after	13.66	12.87	14.46	1.49	0.96
AG – before	20.21	18.02	22.40	4.11	0.11
AG – after	14.33	12.59	16.06	3.26	0.10

M – mean; Me – median, SD – standard deviation, p S-W – p values for Shapiro-Wilk test.

**FIG. 2:**
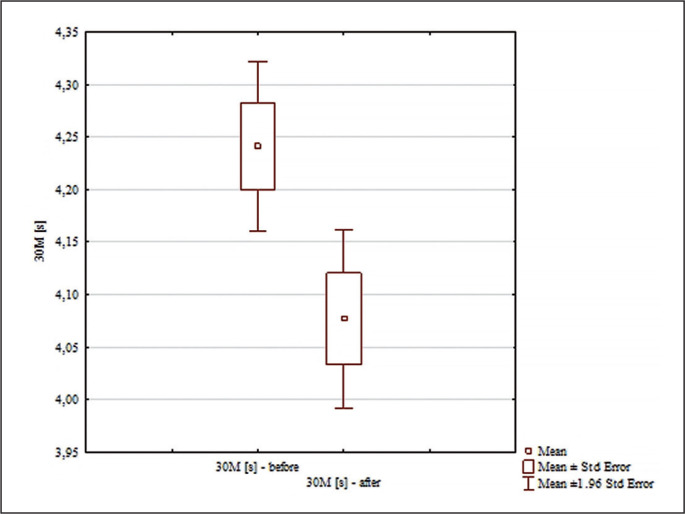
Comparisons between paired values for 30 m [s].

The analysis of the results for the 30 m [s] distance presented in Figure 2 showed significant differences for the main effects only for the pre-post effect (F = 44.20; p < 0.0001; ɳ^2^ = 0.59); and for the interaction group x measurement time (F = 15.54; p = 0.00044; ɳ^2^ = 0.34). No significant differences were found between the results of the 30 m [s] test in the analyzed groups before the experiment (p = 0.99). Significant differences were found only between the results before and after in the experimental group (p = 0.0002; d = 0.97). In the experimental group, the time needed to run a distance of 30 m was statistically significantly shorter after the experiment (Fig. 2).

**FIG. 3:**
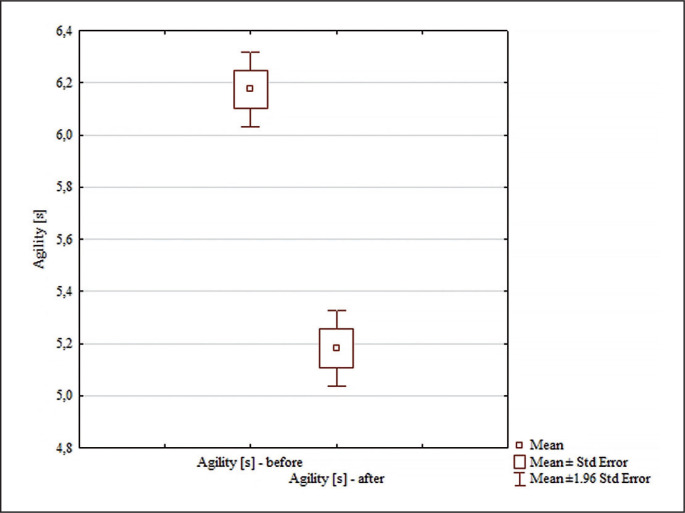
Comparisons between paired values for agility [s].

The analysis of the agility test results included in Figure 3 revealed significant differences for the main effects: differences between groups (F = 36.95; p = < 0.0001; ɳ^2^ = 0.55); differences for the main effects before and after (F = 150.94; p < 0.0001; ɳ^2^ = 0.83); and for the interaction group x measurement time (F = 91.15; p < 0.0001; ɳ^2^ = 0.75). No significant differences were found between the pre-test agility test results [s] in the analyzed groups (p = 0.96). Significant differences between pre and post results were found only in the experimental group (p = 0.0001; d = 3.41). In the experimental group, the time required to perform the agility test was statistically significantly shorter after the experiment (Fig. 3). Significant differences were found between the experimental and control groups after the experiment (p = 0.0001; d = 3.42). After the experiment, the time required to perform the agility test was statistically significantly shorter in the experimental group than in the control group (Fig. 3).

**FIG. 4:**
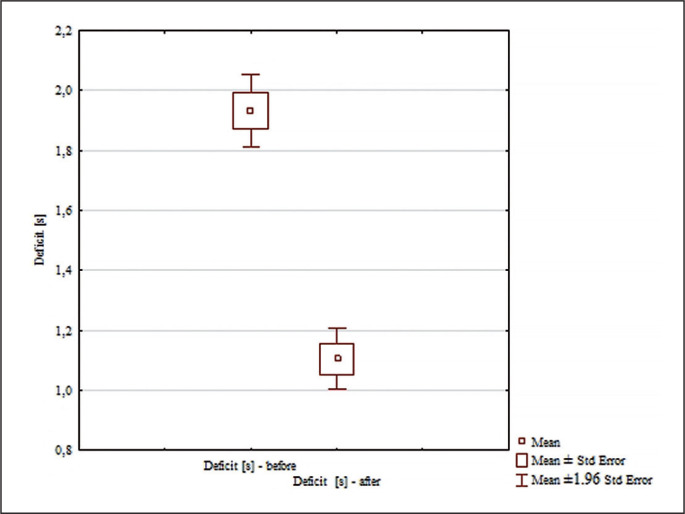
Comparisons between paired values for deficit [s].

Analysis of the “Deficit” scores in Figure 4 revealed significant differences for the main effects: differences between groups (F = 62.98; p < 0.0001; ɳ^2^ = 0.67); differences for the main effects before and after (F = 125.21; p < 0.0001; ɳ^2^ = 0.81); and for the interaction group x measurement time (F = 83.95; p < 0.0001; ɳ^2^ = 0.74). No significant differences were found between the pre-test deficit scores of the groups analyzed (p = 0.80). Significant differences between the pre and post scores were found only in the experimental group (p = 0.0002, d = 1.61). Deficit was statistically significantly lower in the experimental group after the experiment (Fig. 4). Significant differences were found between the experimental and control groups after the experiment (p = 0.00016; d = 4.33). The deficit was statistically significantly lower in the experimental group than in the control group after the experiment (Fig. 4).

**FIG. 5:**
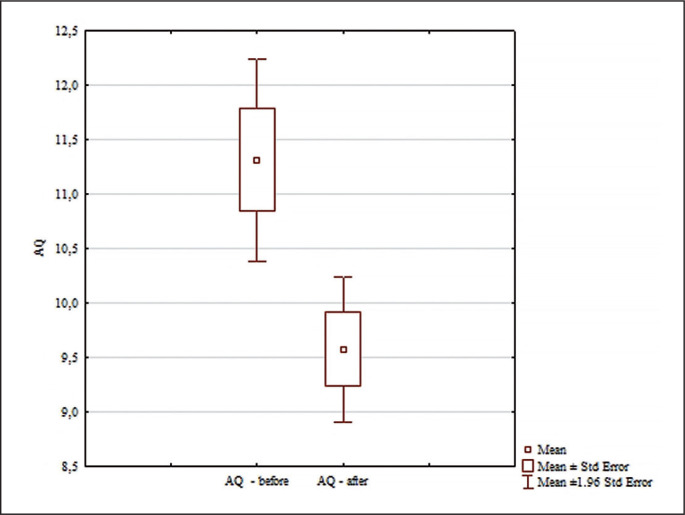
Comparisons between paired values for AQ.

The analysis of the results for AQ included in Figure 5 revealed significant differences for the main effects only for the pre and post effect (F = 10.36; p = 0.0031; ɳ^2^ = 0.26); and for the interaction group x measurement time (F = 5.02; p = 0.032; ɳ^2^ = 0.14). No significant differences were found between the pre-test AQs of the analyzed groups (p = 0.99). Significant differences were found only between pre/post results in the experimental group (p = 0.0031; d = 1.05). In the experimental group, the AQ score was statistically significantly lower after the experiment (Fig. 5).

**FIG. 6:**
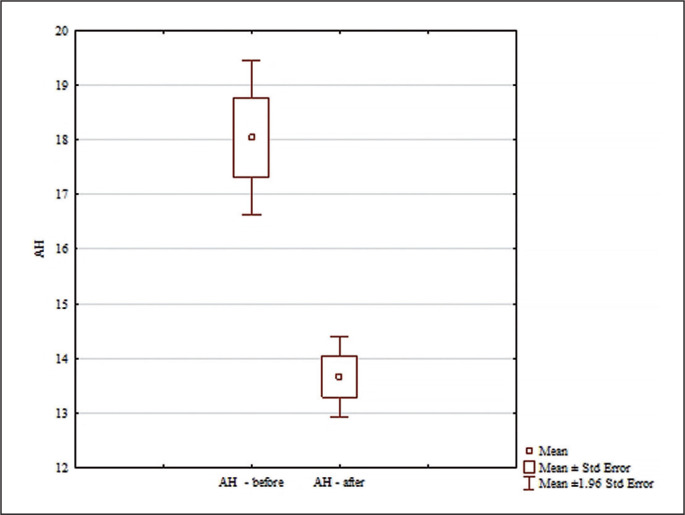
Comparisons between paired values for 5 m AH.

The analysis of the AH results included in Figure 6 showed significant differences for the main effects: differences between groups (F = 7.65; p = 0.009; ɳ^2^ = 0.20); differences for the main effects for pre and post conditions (F = 32.97; p < 0.0001; ɳ^2^ = 0.52); and for the interaction group x measurement time (F = 18.11; p = 0.00018; ɳ^2^ = 0.38). No significant differences were found between the AH results of the groups analyzed before the experiment (p = 0.99). Significant differences between pre/post results were found only in the experimental group (p = 0.00016; d = 1.91).In the experimental group, AH scores were statistically significantly lower post experiment (Figure 6). Significant differences were found between the experimental and control groups after the experiment (p = 0.00025; d = 2.06). After the experiment, the AH score in the experimental group was statistically significantly lower than in the control group (Fig. 6).

**FIG. 7:**
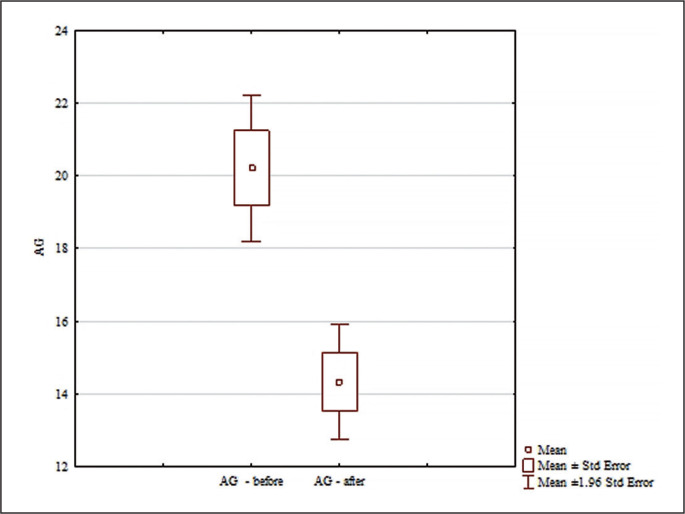
Comparisons between paired values for AG.

Analysis of the AG results in Fig. 7 found significant differences for the main effects: differences between the groups (F = 4.45; p = 0.043; ɳ^2^ = 0.13) ; differences for the main effects before and after (F = 130.76; p < 0.0001; ɳ^2^ = 0.81) ; and for the interaction group x measurement time (F = 106.88; p < 0.0001; ɳ^2^ = 0.72). No significant differences were found between the AG results in the analyzed groups (p = 0.99) before the experiment. Significant differences were found between the results before and after only in the experimental group (p = 0.0001; d = 1.59) In the experimental group, AG scores were statistically significantly lower after the experiment (Fig. 7). Significant differences were found between the experimental and control groups after the experiment (p = 0.0016; d = 1.56). After the experiment, the AG score in the experimental group was statistically significantly lower than in the control group (Fig. 7).

## DISCUSSION

Speed and agility are the result of multiple anatomical systems working together in highly coordinated manner, including the cross-body co-contractions of deep myofascial tissues that create core stiffness; the pulsing contract-and-release cycle of the nervous system that efficiently generates rapid, powerful ground contact; and the hormonal reactions that produce adrenaline, cortisol, and lactic acid. A comprehensive approach via combining speed, agility, and strength training significantly improved the results in all of the tested motor variables.

The data analysis showed significant improvement in 5 m and 30 m sprints after applying the 8-week targeted training protocol.

A statistically significant improvement in performance was also observed after the 8-week training period in case of COD, while the speed deficit also decreased significantly. The greatest improvements were observed during the COD test, what can best translate in soccer players into actions on the pitch. As a result of repeated COD training over a period of 8 weeks, its technique was improved. The changes focused on maintaining a low athletic stance with slight flexion at the hips, knees, and ankles with joint angles adjusted to allow for optimal power transfer and rapid re-acceleration.

The control group also achieved a statistically significant improvement in most of the motor variables tested, with the exception of 30 m, deficit, AQ, AH, AG. However, these changes were minor compared to the experimental group.

Maintaining the lowered position, also known as the athletic stance, is crucial for cutting, sidestepping, pivoting, and other COD maneuvers during the match. The mechanics of COD can be separated into three phases: deceleration when the muscles primarily contract eccentrically, transition when the muscles primarily contract isometrically and reacceleration when concentric contractions of the muscles dominate. The 8 weeks of targeted strength training significantly translated into improvements in COD.

Significant differences were registered in the reduction of asymmetry between the muscle groups studied before and after the 8-week training intervention. The greatest effect was obtained for the gluteal muscles. Lower levels of asymmetry significantly translate into a reduced risk of injury and allow athletes to move cost-effectively. Movement patterns are the result of typical everyday activities, habits, leg dominance, and a history of injuries [23, 24]. A side-to-side asymmetry greater than 15% [15] may increase the risk of knee injuries. Soccer players are frequently characterized by unequal development of the right and left limbs due to certain technical actions performed in the sport, and such disproportions can cause functional or even structural asymmetries. In the present study, an 8-week specific movement pattern resistance training had a statistically significant effect on the reduction of these disproportions in the quadriceps, hamstring, and gluteal muscles, which may translate into a reduction of injury risk. The literature on muscle activity during running lacks data on the changes in the pattern of muscle activity following a specific period of resistance training. Furthermore, no studies have demonstrated differences in the activity of particular muscles and their comparison with each other. Comparisons of overall muscle activity of the right and left limbs often fail to show disproportions, whereas analysis of particular muscles with respect to each other (AQ, AH, AG) reveals significant statistical differences. This may result from the fact that the central nervous system compensates for the disturbances in the activity of particular muscles by transferring part of the load to other muscles. Consequently, the activity of the right lower limb can be taken over and controlled by a different muscle than in the left limb. Training based on movement patterns and activation of particular muscles can reduce the disproportion of (AQ, AH, AG) with respect to each other.

Most soccer players have a preferred foot for kicking the ball, and it is believed that this preference may lead to an asymmetry in the strength and flexibility of the lower extremities. Daneshjoo [25] attempted to determine whether asymmetry in strength and flexibility is present in the lower limbs of soccer players. They found significant differences between the preferred and non-preferred leg in the knee flexors and for the dynamic control ratio [26]. In both cases, the knee flexors of the preferred leg were weaker than those of the non-preferred leg. Almost 68% of players had significant musculoskeletal abnormalities (imbalance > 10%) in one or more specific muscle groups, while no significant differences were found in flexibility of the hip joint between the preferred and non-preferred leg. Our results indicate that the greatest disproportion between the right and left side occurs in the glute muscles and the smallest in the quadriceps muscles. These results indicate a greater need for activating the glute and hamstring muscles during resistance training for soccer players.

## CONCLUSIONS

Implementation of training methods, which target specific aspects of soccer in the pre-season training routines can improve key motor abilities for that sports discipline. A comprehensive training approach including speed, agility, and resistance training based on specific movement patterns should be applied by strength and conditioning practitioners in soccer teams to achieve peak physical performance and reduce injury risk due to the partial elimination of muscular asymmetries. Future research should focus on other components of training, such as plyometrics with regard to muscle activity patterns to develop optimal pre-season training programs for soccer players. Different age, sex and competition levels should be considered as well.

## Declarations

The research project was approved by the Committee of Bioethics at the Academy of Physical Education in Katowice.
